# Prolonged mechanical ventilation worsens sepsis-induced diaphragmatic dysfunction in the rat

**DOI:** 10.1371/journal.pone.0200429

**Published:** 2018-08-01

**Authors:** Matthieu Le Dinh, Serge Carreira, Julie Obert, Ghislaine Gayan-Ramirez, Bruno Riou, Maud Beuvin, Thomas Similowski, Catherine Coirault, Alexandre Demoule

**Affiliations:** 1 Sorbonnes Universités UPMC Univ Paris 06, UMRS INSERM 1158, Paris, France; 2 Department of Anesthesiology and Critical Care Groupe hospitalier Pitié-Salpêtrière, Assistance Publique-Hôpitaux de Paris (APHP), Paris, France; 3 Sorbonnes Universités UPMC Univ Paris 06, UMRS INSERM 1166, IHU ICAN, Paris, France; 4 Sorbonnes Universités UPMC Univ Paris 06, UMRS INSERM 974, Institut de Myologie, Paris, France; 5 Respiratory Muscle Research Unit, Laboratory of Pneumology and Respiratory Division, Katholieke Universiteit, Leuven, Belgium; 6 Department of Emergency Medicine and Surgery Groupe hospitalier Pitié-Salpêtrière, APHP, Paris, France; 7 Department of Pneumology and Medical Intensive Care Groupe hospitalier Pitié-Salpêtrière, APHP, Paris France; National Yang-Ming University, TAIWAN

## Abstract

**Background:**

Short-term mechanical ventilation (MV) protects against sepsis-induced diaphragmatic dysfunction. Prolonged MV induces diaphragmatic dysfunction in non-septic animals, but few reports describe the effects of prolonged MV in sepsis. We hypothesized that prolonged MV is not protective but worsens the diaphragmatic dysfunction induced by a mild sepsis, because MV and sepsis share key signaling mechanisms, such as cytokine upregulation.

**Method:**

We studied the impact of prolonged MV (12 h) in four groups (n = 8) of male Wistar rats: 1) endotoxemia induced by intraperitoneal injection of *Escherichia coli* lipopolysaccharide, 2) MV without endotoxemia, 3) combination of endotoxemia and MV and 4) sham control. Diaphragm mechanical performance, pro-inflammatory cytokine concentrations (Tumor Necrosis Factor-α, Interleukin-1β, Interleukin-6) in plasma were measured.

**Results:**

Prolonged MV and sepsis independtly reduced maximum diaphragm force (-27%, P = 0.003; -37%, P<0.001; respectively). MV and sepsis acted additively to further decrease diaphragm force (-62%, P<0.001). Similar results were observed for diaphragm kinetics (maximum lengthening velocity -47%, P<0.001). Sepsis and MV reduced diaphragm cross sectional area of type I and IIx fibers, which was further increased by the combination of sepsis and MV (all P<0.05). Sepsis and MV were individually associated with the presence of a robust perimysial inflammatory infiltrate, which was more marked when sepsis and MV were both present (all P<0.05). Sepsis and, to a lesser extent, MV increased proinflammatory cytokine production in plasma and diaphragm (all P<0.05); proinflammatory cytokine expression in plasma was increased further by the combination of sepsis and MV (all P<0.05). Maximum diaphragm force correlated negatively with plasma and diaphragmatic cytokine production (all p<0.05).

**Conclusions:**

Prolonged (12 h) MV exacerbated sepsis-induced decrease in diaphragm performance. Systemic and diaphragmatic overproduction of pro-inflammatory cytokines may contribute to diaphragm weakness.

## Introduction

Severe sepsis causes diaphragm dysfunction and subsequent ventilatory pump failure [1], which contributes to the respiratory failure observed in a large proportion of septic patients [2, 3]. Mechanical ventilation (MV) is often required in this clinical setting. In addition to sustaining gas exchange and therefore constituting life-saving therapy, short-term (4-h) MV protects against sepsis-induced diaphragmatic dysfunction [4]. However, the clinical relevance of this short-term protection is questionable, as septic patients commonly require longer durations of MV [5]. Moreover, as 12 h of MV induces contractile dysfunction known as ventilator-induced diaphragmatic dysfunction in both patients and healthy non-septic animals [6–9], it is reasonable to hypothesize that sepsis and prolonged MV could have an additive if not a synergic deleterious impact on diaphragmatic function. In support of this hypothesis, sepsis-induced diaphragm dysfunction and ventilator-induced diaphragm dysfunction share key signalling mechanisms. Both of these processes involve pro-inflammatory cytokine upregulation [10, 11] and oxidative stress [12–15] that may in turn depress diaphragmatic function [16]. However, few data are available concerning the interaction between sepsis and more prolonged MV. Two recent studies addressed this issue. The first one showed that sepsis did not affect ventilator-induced diaphragm dysfunction [17], but sepsis was very mild in this study. The second one showed that MV instituted after 12 hours of a severe sepsis turning to septic shock severely worsened ventilator induced diaphragm dysfunction [18]. In the present study, we hypothesized that a MV instituted at induction of a mild sepsis would worsen sepsis-induced diaphragmatic dysfunction. To avoid the devastating effect of septic shock on survival in mechanically ventilated animals, we used a previously described model of mild endotoxemia [19]. We also hypothesized that this deleterious effect could be mediated by a further increase in proinflammatory cytokine production levels in plasma and in the diaphragm, since these mediators are both involved in sepsis-induced and ventilator-induced diaphragm dysfunction.

## Materials and methods

Experiments were conducted in an authorized laboratory under supervision of an authorized researcher (C. Coirault, A-75-786). The project was approved by the relevant Animal Care Committee through the French Ministry of High Education and Research (Comité Régional d’Ethique en Expérimentation Animale Paris–Comité 3, Paris, France). Animal care and handling were performed in accordance with the Guidelines of the Institutional and Animal Care and Use Committees in an authorized laboratory (agreement number B 75-13-08).

Adult male Wistar rats (450 to 600 g; Charles River Laboratories, L’Arbresle, France) were randomly assigned to one of the following four groups: (1) spontaneously breathing endotoxemic group (SV-LPS group, n = 8); (2) mechanically ventilated group (MV group, n = 8); (3) mechanically ventilated endotoxemic group (MV-LPS group, n = 8); (4) spontaneously breathing sham control group (Control group, n = 8). Each procedure was performed for a 12-h period.

### Experimental procedures

Mechanically ventilated rats (MV and MV-LPS groups) were anesthetized with sodium pentobarbital (40 mg.kg^-1^ intraperitoneally) and tracheotomized. During the entire experiment, animals were ventilated with a volume-driven small animal ventilator (Model 665A, Harvard Apparatus, Holliston, MA). The tidal volume was set at 0.5 mL/100 g body weight and respiratory rate was set at 55–60 breaths/min. Breathing air was humidified and enriched with oxygen. Ventilator settings and oxygen concentration were adjusted to maintain PaCO_2_ between 35 and 40 mmHg and PaO_2_ between 80 and 100 mmHg. Airway pressure was monitored continuously to ensure complete relaxation of the diaphragm under MV (DP15-32, Validyne, Northridge, CA). The right carotid artery was cannulated and connected to a pressure transducer to monitor arterial blood pressure and heart rate (Blood pressure transducer TD104A, Biopac systems, Santa Barbara, CA). The tail vein was cannulated for continuous infusion of isotonic saline at a rate of 1 mL.h^-1^ regardless of weight (Baxter, Deerfield, IL). During the experiments, continuous infusion of heparin (10 IU.h^-1^, Sanofi, Paris, France) was administered via the carotid artery and pentobarbital (CEVA Animal Health, Libourne, France) was administered via the tail vein using a pump (Pilote A2, Fresenius Kabi, Bad Homburg, Germany) at an initial dose of 0.5 mg.h^-1^. Body temperature was maintained at 37°C during the entire experiment by external warming with a homoeothermic blanket system (Harvard Apparatus).

In the endotoxemic groups (SV-LPS and VM-LPS groups), 1 mL of saline solution containing lipopolysaccharide (1 mg.kg^-1^, *Escherichia coli* serotype O111:B9, Sigma Chemical Co, St Louis, MO) was administered intraperitoneally [19]. In the VM-LPS group, LPS was administered at the onset of MV. Sham control and MV groups were submitted to the same procedure with an equivalent 1 mL volume of saline.

Arterial blood gases and plasma lactate level were determined at 6 h in the MV and MV-LPS animals and at the end of the 12-h protocol in all four groups (GEM Premier 3000 Critical Care Analyser, Instrumentation Laboratory, Saint-Mandé, France).

At the end of the 12-h protocol, animals received an injection of 40 mg.kg^-1^ pentobarbital. A muscle strip from the ventral part of the right and left costal diaphragm was carefully dissected *in situ* for the purposes of contractile function study (see below). Part of the remaining muscle was frozen in liquid nitrogen. Blood was also sampled, centrifuged and plasma was frozen for further measurements. Sacrifice resulted from exsanguination during the dissection of the diaphragm in a previously anesthetized animal (injection of 40 mg.kg^-1^ pentobarbital).

### Diaphragm muscle mechanical properties

Each muscle strip was rapidly mounted in a tissue chamber containing Krebs-Henseleit solution: 118 mM NaCl, 4.7 mM KCl, 1.2 mM MgSO_4_, 1.1 mM KH_2_PO_4_, 25 mM NaHCO_3_, 2.4 mM CaCl_2_ and 5.5 mM glucose. The solution was bubbled with a gas mixture of 95% O_2_−5% CO_2_ and maintained at 27°C and pH 7.4. Muscle extremities were held in spring clips and attached to an electromagnetic force transducer, as previously described [20, 21]. Diaphragm muscle strips were electrically stimulated in twitch and tetanus by means of two silver electrodes positioned parallel to the muscle and delivering electrical stimulation lasting 1 ms. Muscle strips recovered their optimal mechanical performance after a 20 min equilibration period. Mechanical variables were measured at the apex of the length-active isometric force curve (L_max_). Strips were stimulated in twitch and then sequentially in tetanus with trains of 1, 25, 50, 75 and 100 Hz stimuli, and force and shortening were recorded. At the end of the experiment, each muscle cross-sectional area (in mm^2^) was calculated from the ratio of muscle weight to muscle length at L_max_, assuming a muscle density of 1.06. All analyses were performed from digital records of force and length obtained with a computer. Conventional mechanical variables at L_max_ were calculated from three contractions. The first contraction was isotonic and was loaded with the preload corresponding to L_max_. The second contraction was abruptly clamped to zero load just after the electrical stimulus with critical damping. The third contraction was fully isometric at L_max_. In all groups, maximum tetanic isometric force was achieved at a stimulation frequency of 100 Hz with train duration of 250 ms. Maximum lengthening velocity (VL_max_) was measured from contraction 1 and maximum unloaded shortening velocity (V_max_) was measured from contraction 2. Maximum isometric force normalized per cross-sectional area, the positive peak of the force derivative (+dF.dt^-1^), and the negative peak of the force derivative (-dF.dt^-1^) were measured from contraction 3. Maximum isometric force normalized per cross-sectional area was also measured in response to twitch stimulation.

### Histologic and morphologic analysis

Serial sections of the costal diaphragm were cut at 10-μm thickness with a cryostat kept at -20°C. Sections were stained with hematoxylin and eosin and with Oil Red O and were analyzed qualitatively for structural abnormalities by an expert unaware of the study experimental design. Pixel intensity based thresholding was used to quantify the percentage of ORO positive fibers. Other sections were stained with either mouse anti-slow myosin heavy chain (MyHC-1) or anti-fast myosin heavy chain (MyHC-2a and MyHC-2x). Cross-sectional areas of each fiber type were determined from immunofluorescence of myosin heavy chain isoforms using a Olympus FV 100 (Olympus, Hamilton, Bermuda) microscope at x20 magnification, and then analyzed using ImageJ software (version 1.51).

### Cytokine production levels

Frozen diaphragm samples were homogenized with Triton-HEPES buffer (1% Triton X-100, 50 mM HEPES (pH 8); 150 mM NaCl, 10% glycerol, 2 mM EDTA; 1.5 mM MgCl_2_, 10 IU.mL^-1^ protease inhibitor cocktail (Sigma Aldrich). Total protein was determined using a BCA assay method (BioRad Laboratories, Hercules, CA). Levels of tumor necrosis factor (TNF)-α, interleukin (IL)-1β and IL-6 in the diaphragm and in plasma were measured by enzyme-linked immunosorbent assay kits (HS Quantikine; R&D Systems, Minneapolis, MN).

### Statistical analysis

Data are expressed as median (25–75 interquartile). Between-group comparisons were performed with one-way or repeated measure two way analysis of variance followed, when significant, by a post-hoc Tukey test for normally distributed data (Kolmogorov-Smirnov test) and a Kruskal-Wallis test followed by Dunn’s post-hoc test for non-normally distributed data. The effect of sepsis and MV on isometric peak force of the diaphragm was evaluated using two-way (group effect and stimulation frequency effect) analysis of variance. Correlations were performed with the Spearman test in order to be as conservative as possible. We determined that a sample size of n = 8 per group would enable us to detect a 35% decrease in force assuming a baseline value of force of 93 ± 18 mN.mm^-2^ [21], an alpha risk of 0.05 and a beta risk of 0.20, (PASS 11 software, Statistical Solutions Ltd., Cork, Ireland). All statistical analyses were performed using GraphPad 5 (GraphPad Software, La Jolla, CA). All P values were two-tailed and a P value <0.05 was considered significant.

## Results

Arterial blood pressure at the end of the 12-h protocol was significantly lower in MV-LPS animals than in Control and LPS animals (Table 1). Animals that received LPS demonstrated higher plasma lactate levels and subsequent metabolic acidosis, as indicated by decreased bicarbonate levels (Table 1)_._ In the SV-LPS group, this lactic acidosis was compensated by a lower PaCO_2_, consistent with the presence of hyperventilation. In contrast, MV-LPS group demonstrated a significantly lower pH compared to Controls (Table 1). Blood gases and lactate levels at 6 h are displayed in S1 Table.

**Table 1:** Mean arterial blood pressure and arterial blood gases at the end of the 12-h protocol.

	Control (n = 8)	MV (n = 8)	SV-LPS (n = 8)	MV-LPS (n = 8)
Mean ABP, *mmHg*	135 (126, 151)	118 (103, 136)	140 (135, 158)	85 (63, 100)*^$^
pH	7.38 (7.34, 7.39)	7.37 (7.28, 7.44)	7.42 (7.39, 7.46)	7.25 (7.15, 7.32)*^†^^$^
PaCO_2_, *mmHg*	42 (38, 46)	39 (33, 52)	33 (29, 40)	42 (36, 46)
PaO_2_, *mmHg*	113 (92, 142)	89 (69, 127)	88 (81, 94)	124 (82, 150)
HCO_3_^-^, *mM*	23.5 (21.8, 27.2)	20.9 (19.7, 23.6)	21.7 (18.7, 25.7)	17.5 (14.5, 21.6)
Lactate, *mM*	1.2 (0.9, 1.4)	1.6 (1.4, 4.9)	3.3 (2.7, 5.5)*	2.9 (2.7, 3.9) *

Data are median (interquatile range). Control = spontaneous ventilation without endotoxemia; MV = mechanical ventilation without sepsis; SV-LPS = endotoxemia with spontaneous ventilation; MV-LPS = endotoxemia with mechanical ventilation; ABP, mean arterial blood pressure; PaCO_2_ = arterial partial pressure of carbon dioxide; PaO_2_ = arterial partial pressure of oxygen; HCO_3_^-^ = bicarbonate.

*: P<0.05 versus Control

†: P<0.05 versus MV

$: P<0.05 versus SV-LPS.

### Diaphragm contractility

**Fig 1:**
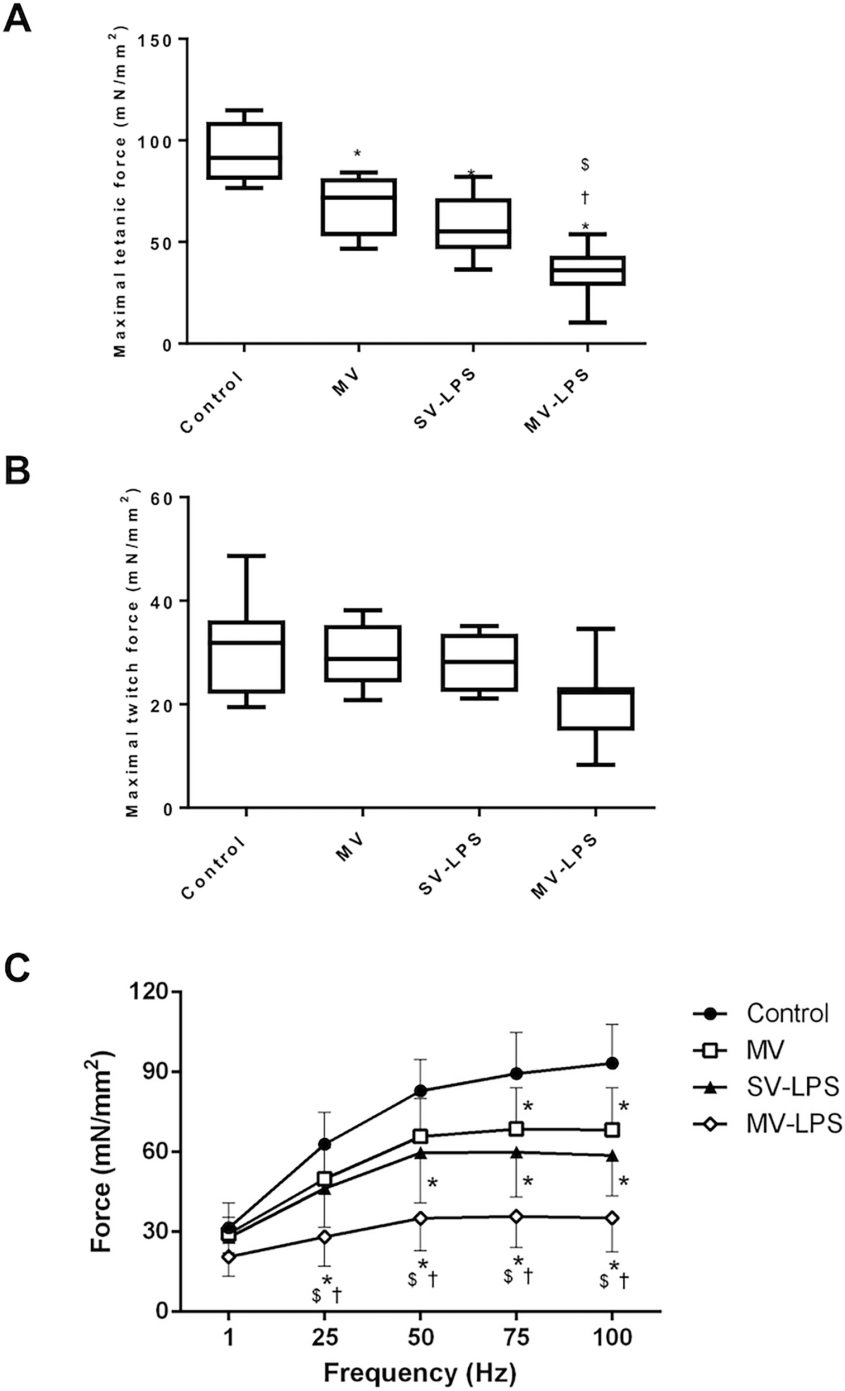
**Maximal tetanic force (Panel A), maximal twitch force (Panel B) and force-frequency relationship (Panel C) of the diaphragm in the four experimental groups.** Control = spontaneous ventilation without endotoxemia; MV = mechanical ventilation without sepsis; SV-LPS = endotoxemia with spontaneous ventilation; MV-LPS = endotoxemia with mechanical ventilation. Results are presented in the form of box plots. Boxes are drawn between the first and third quartiles of the distribution, black bars indicate the median, and whiskers indicate the minimum and maximum values, n = 8 per group. *: P<0.05 versus Control; †: P<0.05 versus MV; $: P< 0.05 versus SV-LPS.

Both mechanical ventilation and sepsis affected the maximal tetanic force production as attested by a reduction of 100 Hz tetanic force in MV and SV-LPS groups (-27% and -37%, respectively, p = 0.017 and p = 0.0002) (Table 2). However, the MV-LPS group exhibited the most severe muscle weakness, with a 62% (p<0.0001) reduction in 100 Hz maximum tetanic force compared to Controls. A significant interaction was observed between stimulation frequency and group (F = 2.85, P = 0.002), indicating that the impact of the experimental group on diaphragm strength varied according to the stimulation frequency. Twitch force followed the same patterns of change as those reported for tetanus. This marked reduction in force production was also present over the entire range of tetanic stimulation frequencies studied in the MV-LPS group (Fig 1). Diaphragmatic kinetic properties were significantly different only in the MV-LPS group, as V_max_, VL_max_ and +dF.dt^-1^ were not significantly different between groups, except in the MV-LPS group, where V_max_, VL_max_ and +dF.dt^-1^ were slower than in Controls (-34%, -48% and -51%, respectively) (Table 2). Finally, compared to controls, -dF.dt^-1^ was not decreased in the MV group but was significantly decreased in the SV-LPS group (-41%) and further decreased in the MV-LPS group (-67%) (Table 2).

**Table 2:** Diaphragmatic contractile properties.

	Control (n = 8)	MV (n = 8)	SV-LPS (n = 8)	MV-LPS (n = 8)
Total force, twitch (mN.mm^-2^)	32 (22, 36)	29 (24, 35)	28 (22, 34)	22 (15, 23) *
Total force, 100 Hz (mN.mm^-2^)	91 (81, 109)	72 (53, 81) *	55 (46, 72) *	36 (28, 43) *^†^^$^
VL_max_, 100 Hz (L_max_.s^-1^)	2.9 (2.7, 3.2)	3.0 (2.6, 3.2)	2.6 (1.9, 3.0)	1.6 (0.9, 2.2) *^†^^$^
V_max_, 100 Hz (L_max_.s^-1^)	4.8 (4.6, 5.2)	4.9 (4.8, 4.9)	4.8 (3.7, 5.0)	3.3 (2.4, 4.0) *^†^^$^
+dF.dt^-1^, 100 Hz (mN.s^-1^)	1125 (999, 1436)	1159 (884, 1492)	899 (765, 1208)	627 (408, 783) *^†^
-dF.dt^-1^, 100 Hz (mN.s^-1^)	-1201 (-1528, -1084)	-1043 (-1274, -670)	-705 (-966, -523) *	-408 (-589, -309) *^†^

Data are median (interquatile range). Control = spontaneous ventilation without endotoxemia; MV = mechanical ventilation without sepsis; SV-LPS = endotoxemia with spontaneous ventilation; MV-LPS = endotoxemia with mechanical ventilation. L_max_ = resting length; VL_max_ = maximum lengthening velocity (isotonic contraction); V_max_ = maximal unloaded shortening velocity (zero load clamp); +dF.dt^-1^ = peak positive force derivative normalized per cross-sectional area; -dF.dt^-1^ = peak negative derivative normalized to cross-sectional area.

*: P<0.05 versus Control

†: P<0.05 versus MV

$: P<0.05 versus SV-LPS.

### Histologic and histochemical analysis

**Fig 2:**
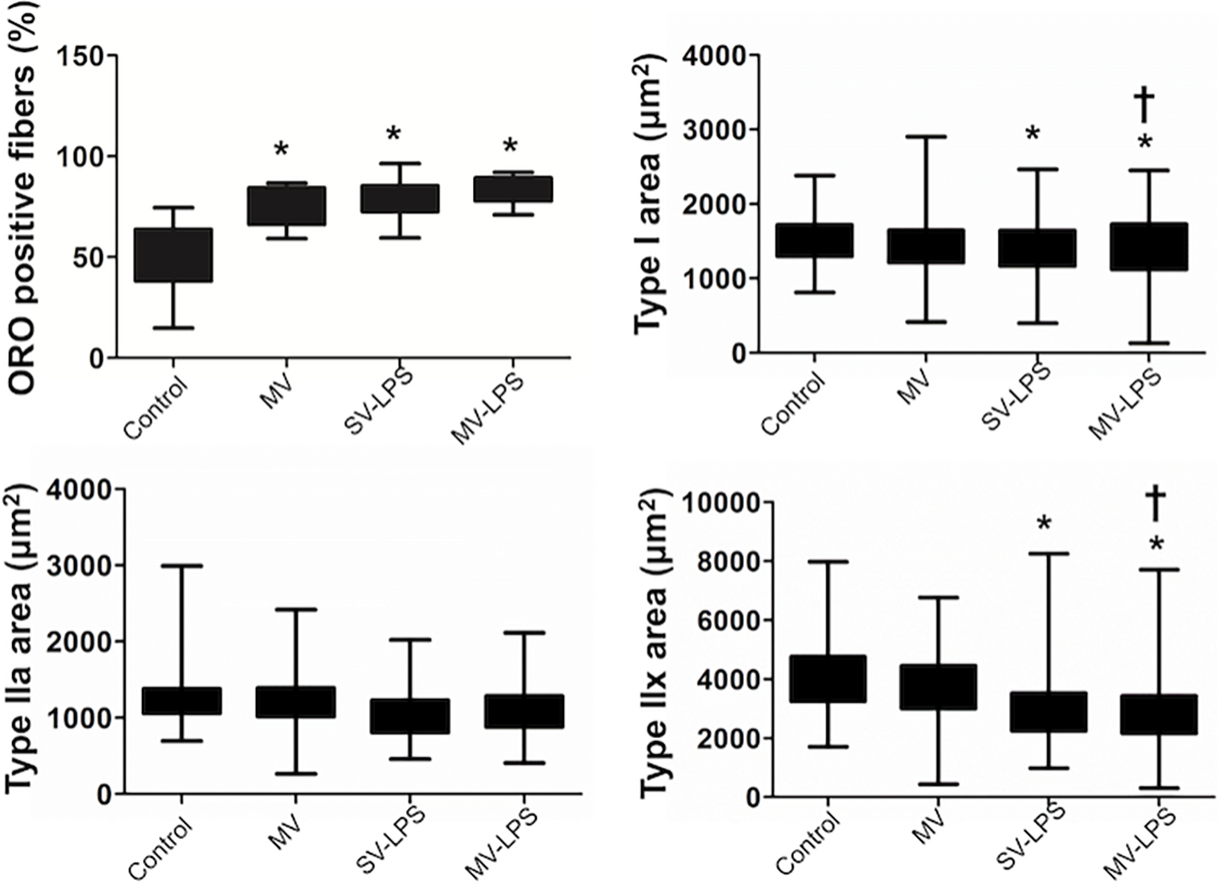
**Proportion of diaphragm myofibers in which intramyocellular fat droplets was observed (panel A) and diaphragm cross-sectional area (*CSA*) of type I (panel B), type IIa, (panel C) and type IIx fibres (panel D) in the four experimental groups.** ORO = Oil Red O; Control = spontaneous ventilation without endotoxemia; MV = mechanical ventilation without sepsis; SV-LPS = endotoxemia with spontaneous ventilation; MV-LPS = endotoxemia with mechanical ventilation. Results are presented in the form of box plots as well as individual values. Boxes are drawn between the first and third quartiles of the distribution, black bars indicate the median, and whiskers indicate the minimum and maximum values, n > 4 per group. *: P<0.05 versus Control; †: P<0.05 versus MV.

Fig 2 depicts structural analysis of the diaphragm (see also S1–S3 Figs). No inflammatory infiltrate was observed in the diaphragm of Controls while a robust perimysial inflammatory infiltrate was observed in the three other groups. This inflammatory infiltrate was qualitatively more marked in the MV-LPS group than in the MV and SV-LPS groups (S1 Fig). Diaphragm intramyocellular fat droplets (i.e., fat located inside muscle fibers, Oil Red O staining) were observed in all groups but were more numerous in the MV, SV-LPS and MV-LPS diaphragms compared to the Controls (Fig 2, see also S2 Fig). Finally, diaphragm CSA of type I and IIx fibers decreased significantly in all groups compared with the control group (Fig 2, see also S3 Fig) and was lower in the MV-LPS group than in the MV group. CSA of type IIa fibers were similar in the four groups.

### Pro-inflammatory cytokine concentrations in the diaphragm and in plasma

**Fig 3:**
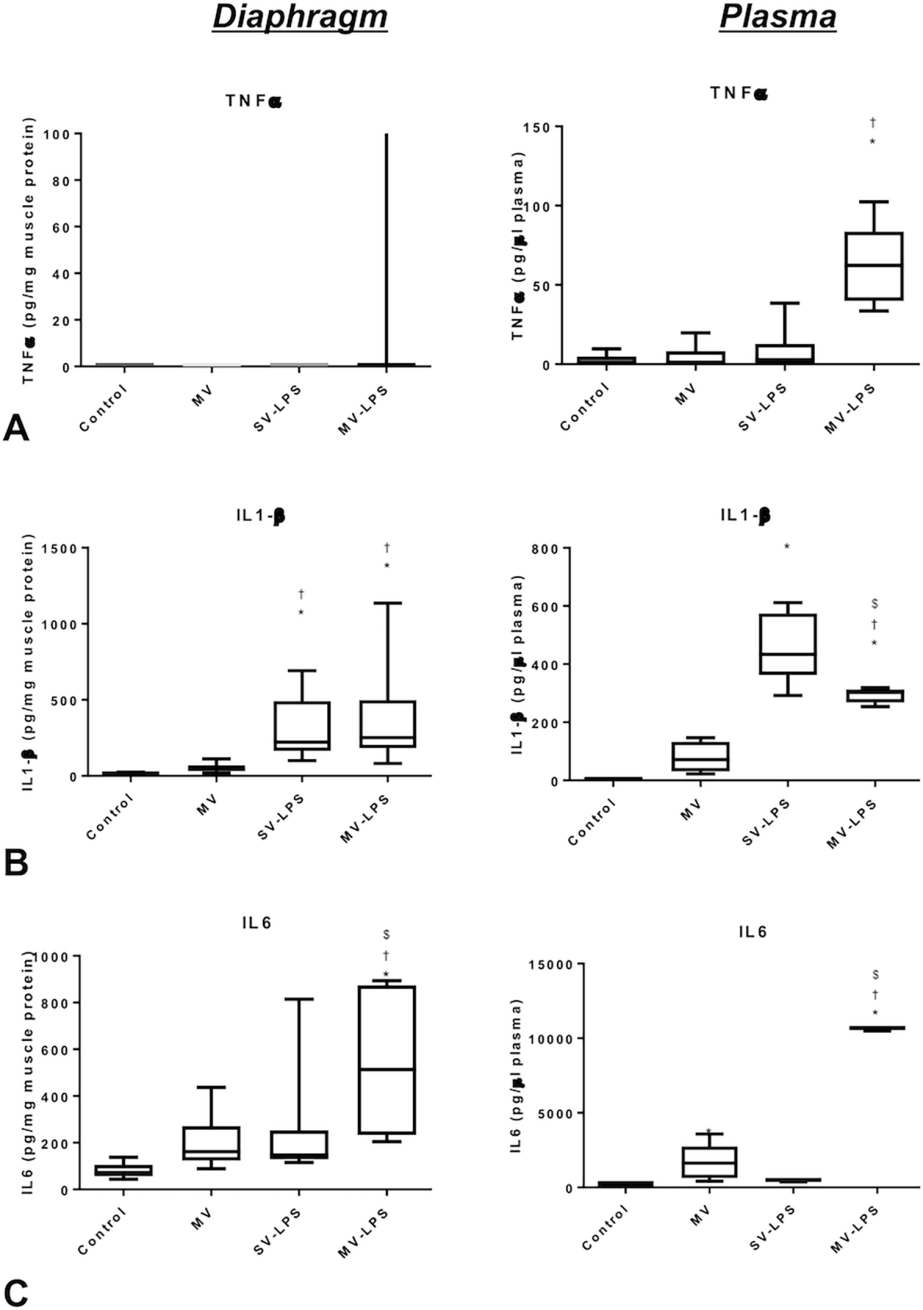
**Tumor necrosis factor (TNF)-α (Panel A), interleukin (IL)-1β (Panel B) and IL-6 (Panel C) protein concentration in the diaphragm (left panels) and in the plasma (right panels) in the four experimental groups.** Control = spontaneous ventilation without endotoxemia; MV = mechanical ventilation without sepsis; SV-LPS = endotoxemia with spontaneous ventilation; MV-LPS = endotoxemia with mechanical ventilation. Results are presented in the form of box plots as well as individual values. Boxes are drawn between the first and third quartiles of the distribution, black bars indicate the median, and whiskers indicate the minimum and maximum values, n = 8 per group. *: P<0.05 versus Control; †: P<0.05 versus MV; $: P< 0.05 versus SV-LPS.

TNF-α was not detected in the diaphragm (Fig 3). IL-1β production in the diaphragm was similarly increased in SV-LPS and LPS-MV animals compared to non-septic groups (Fig 3). IL-6 production was not significantly different between groups, except in the MV-LPS group, where IL-6 production was significantly higher compared to others groups (Fig 3).

The plasma concentration of TNF-α was not significantly different between groups, except in the MV-LPS group, where TNF-α concentration was significantly higher than in the other three groups (Fig 3). Plasma IL-1β concentration was significantly higher in both the SV-LPS and MV-LPS groups than in the other groups (Fig 3). Finally, MV significantly increased plasma IL-6 concentration, which was further increased in the MV-LPS group (Fig 3).

**Fig 4:**
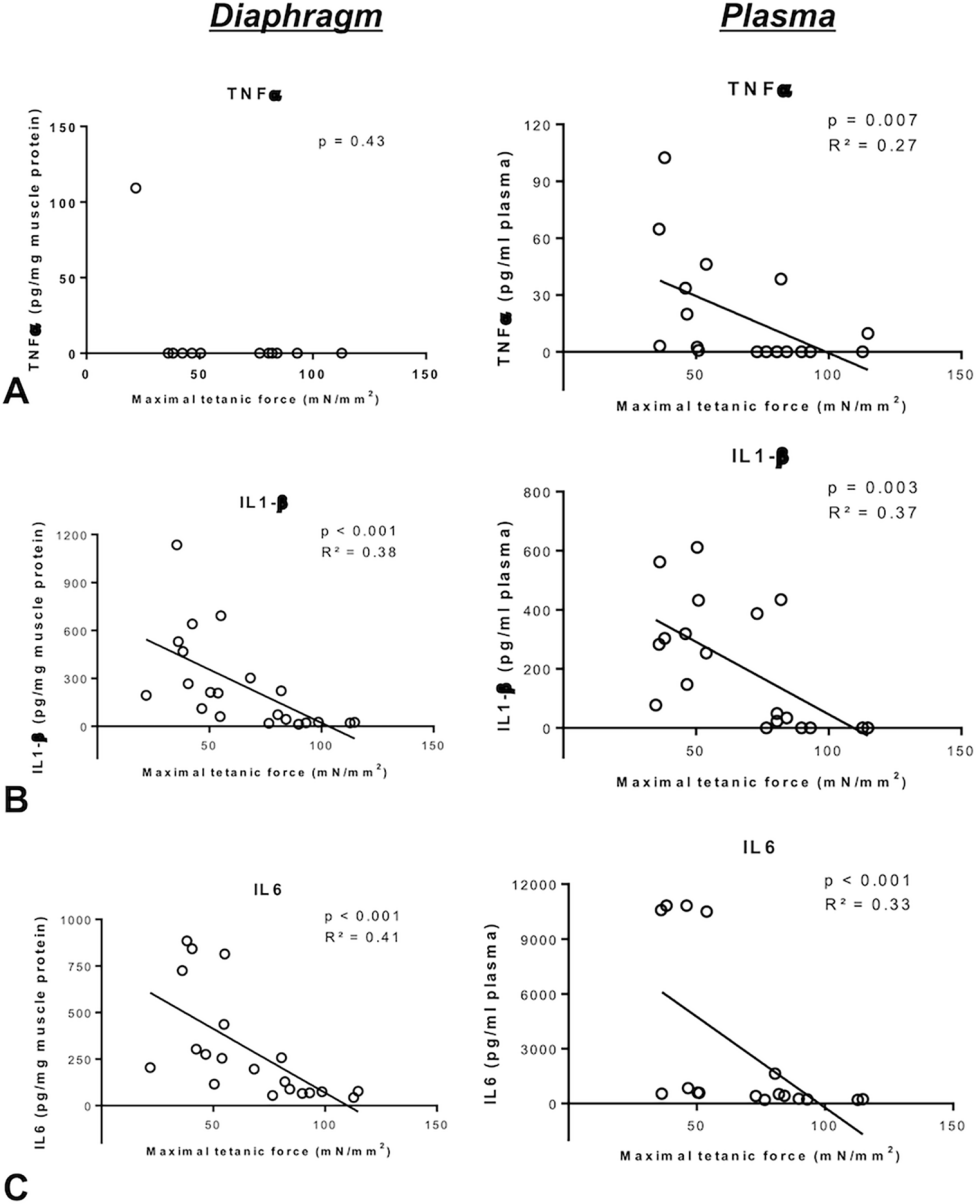
**Relationship between maximum isometric tetanic force of the diaphragm and tumor necrosis factor (TNF)-α (Panel A), interleukin (IL)-1β (Panel B) and IL-6 (Panel C) protein concentration in the diaphragm (left panels) and in the plasma (right panels).** Correlation determined by Spearman test.

Significant negative correlations were demonstrated between maximum diaphragm 100 Hz-tetanic force and diaphragm IL-1β (Fig 4) and IL-6 (Fig 4) protein levels. Similar negative correlations were also observed between maximum diaphragm tetanic force and plasma concentrations of TNF-α, IL-1β and IL-6 (Fig 4).

## Discussion

Our main findings are summarized as follows: (1) prolonged MV and sepsis acted additively to decrease diaphragmatic force production; (2) MV and sepsis both individually induced significant upregulation of plasma proinflammatory cytokine production and acted additively to further increase plasma proinflammatory cytokine production; (3) a similar pattern was observed for proinflammatory cytokine production in the diaphragm; (4) the level of production of proinflammatory cytokines in plasma and in the diaphragm was negatively correlated with diaphragm force production. This study suggests that ventilator-induced diaphragmatic dysfunction is exacerbated by sepsis even in the setting of mild sepsis; upregulation of proinflammatory cytokines contributes to this deleterious effect.

Although sepsis and MV both induce diaphragmatic dysfunction, few data are available concerning the interactions between sepsis and MV. In septic animals, short-term (4 h) MV protects against sepsis-induced diaphragm dysfunction [4]. The mechanism of this protection appears to involve suppression of the deleterious interaction between oxidative and biomechanical stresses on the sarcolemma [4]. To the best of our knowledge, there are only two published report of the interaction between long-term MV and sepsis [17, 18]. In the first report, institution of MV and LPS administration were concomitant and endotoxemia did not enhance ventilator-induced diaphragm dysfunction in piglets nor did it alter myofibre proportion [17]. However, the dose of LPS was much lower (20–30 μg.kg^-1^) than those commonly used in animal models of acute sepsis and information is lacking regarding the severity and duration of the shock and inherent arterial hypotension. Further, blood and diaphragm cytokines were not quantified in this report. In the second study, MV was instituted 12 hours after LPS injection and endotoxemia clearly worsened ventilator induced diaphragm dysfunction [18]. Altered cytokines level of production and myofiber proportion were similar to what we observed. Noticeably, animals undergoing sepsis and MV had a very severe shock. It is therefore impossible to rule out that, in addition to the sepsis, the shock strongly contributed to diaphragm dysfunction. This is why in our study we opted for a model of mild endotoxemia [19]. With this model, we observed an additive deleterious impact of prolonged MV and sepsis on diaphragmatic function. Not only was diaphragm force production altered, but also diaphragm kinetics, as shown by the decrease in many kinetic variables (V_max_, VL_max_, +dF.dt^-1^ and -dF/dt^-1^).

Inflammation plays a major role in diaphragmatic dysfunction in critical illness. Sepsis induces upregulation of multiple proinflammatory genes in the diaphragm, including various proinflammatory cytokines such as TNF-α, IL-1β and IL-6 [10, 22] and there is evidence that these cytokines are involved in sepsis-induced diaphragmatic dysfunction [22]. Moreover, ventilator-induced diaphragmatic dysfunction is also associated with significant upregulation of various proinflammatory cytokines in the diaphragm [11]. In addition, sepsis and, more intriguingly, MV induce an increase in plasma cytokine concentration [11, 23]. In the present study, MV and mild sepsis acted additively to increase both diaphragm and plasma proinflammatory cytokine production in parallel with decreased diaphragm force production.

Many of the proinflammatory cytokines upregulated in the diaphragm by LPS in this study can cause muscle wasting or contractile dysfunction. IL-1 decreases muscle protein synthesis [24]. IL-6 upregulates cathepsin and ubiquitin, two pathways of muscle proteolysis [25] although it may also have beneficial effects on myogenesis through regulation of the proliferative capacity of muscle stem cells on regulation of energy metabolism [26]. TNF-α decreases muscle performance [27], activates protein degradation pathways [28] and destabilizes myogenic transcription factors such as MyoD or myogenin [29, 30]. Overall, the injection an adenoviral vector that overexpresses IL-10 and increases IL-10 serum level, an anti-inflammatory cytokine, significantly inhibits the induction of proinflammatory cytokines in the diaphragm and markedly improves diaphragm force production in infected animals [22].

Our results suggest that prolonged MV is deleterious during sepsis. However, they do not indicate that MV should be avoided in this setting. There is clinical evidence that MV may be required to support the increased work of breathing or for airway protection at the early phase of sepsis [31]. MV also protects against diaphragmatic dysfunction at the early phase of sepsis [4], but is not known whether partial ventilatory support is also protective in the setting of sepsis. However, the present study provided evidence that prolonged controlled MV should be avoided at later stages of sepsis, once the patient’s condition is stable, as currently recommended by the Surviving Sepsis Campaign with the use of weaning protocols [32]. A key challenge consists of finding the best compromise between the early benefit and the late deleterious impact of MV in sepsis. Moreover, our results may also alert the physicians that ventilator-induced diaphragmatic dysfunction may be enhanced in septic patients. Finally, as pro-inflammatory cytokines appear to play an additive role to MV and sepsis to induce diaphragmatic dysfunction, modulation of pro-inflammatory cytokine upregulation could be beneficial. Along these lines, TLR4 knock-out animals exhibit lesser sepsis-induced muscle inflammation [33] and are also less susceptible to ventilator-induced diaphragm dysfunction [11]. Further studies are needed to determine whether modulation of inflammation could be beneficial in this setting.

The following limitations should be considered when assessing the relevance of our results. First, the doses of LPS (1 mg.kg^-1^) used in this model was slightly lower than those commonly used in animal models of endotoxemia, but our preliminary experiments showed that higher doses induced unacceptable mortality rate (>50%). Furthermore, the purpose of this study was to investigate the interaction between MV and sepsis, not septic shock, although arterial blood pressure at 12 hr in the MV-LPS group was 30% less than in the Control group, suggesting that hypotension was also quite severe in this group and not very far from the decrease in arterial blood pressure observed in a previous study [4]. Our results therefore strongly suggest that 12 h of MV worsen sepsis-induced diaphragmatic dysfunction even in the setting of mild sepsis. The duration of MV was also limited to 12 h for similar reasons, as longer durations of MV were associated with high mortality rate. Second this study was performed in the rat and species differences cannot be excluded although we used one of the most commonly used animal models of ventilator-induced and sepsis-induced diaphragmatic dysfunctions. Obviously, one has to keep in mind that 12 h of controlled MV in a rat is quite different from seven days of MV in a patient with periods of controlled and partial MV and the generation of various levels of inspiratory efforts. In this respect, animal models are far from reflecting the high complexity of critical illness in a human being, which involves multiple risk factors. Third, to prevent suffering, animals were anesthetized and we cannot rule out some interference of anesthesia with the sepsis-induced and/or ventilation-induced diaphragmatic dysfunction processes. Fourth, the sham control group consisted of non-tracheostomized rather than tracheostomized rats, since the latter would require sedation and subsequent impaired spontaneous ventilation. Fifth, acidosis is associated with diaphragm dysfunction [34]. As the animals of the MV-LPS group exhibited an uncompensated moderate metabolic acidosis, it is difficult to exclude a direct contribution of acidosis to the diaphragm dysfunction in this group. However, the deleterious impact of uncompensated acidosis on diaphragm function is observed for respiratory acidosis [35] but not in metabolic acidosis [36]. In addition, we evaluated diaphragm contractile properties *in vitro*, after a 20-min washout in a buffer. Of note, although hypotension may contribute to diaphragmatic dysfunction, it is not likely in the present study. Indeed, arterial blood pressure in the MV and MV-LPS group was not different while diaphragmatic dysfunction was more pronounced in the MV-LPS group than in the MV group. Finally, we did not study proteolysis, which has been shown to be involved in ventilator induced diaphragm dysfunction and in sepsis induced diaphragm dysfunction [18].

### Conclusion

Prolonged controlled MV and mild sepsis had an additive deleterious impact on diaphragm performance. Systemic and diaphragmatic overproduction of pro-inflammatory cytokines appeared to contribute to diaphragm weakness in this setting. These results suggest that controlled mechanical ventilation should be avoided; further studies are required to understand whether modalities promoting spontaneous breathing are equally deleterious or alternatively protective in the setting of endotoxaemia. Clinical studies would be useful to determine whether modulation of inflammation and protection offered by contemporaneous modes of ventilatory support are beneficial. Human studies are also needed to evaluate the relevance of these results in MV septic patients.

## Supporting information

S1 Table Arterial blood gases at 6 hours. (PDF)

S1 Fig Hematoxylin and eosin staining of diaphragm. White arrows show inflammatory infiltrates. Control = spontaneous ventilation without endotoxemia; MV = mechanical ventilation without sepsis; SV-LPS = endotoxemia with spontaneous ventilation; MV-LPS = endotoxemia with mechanical ventilation.
Bar scale = 50 μm.
(TIFF)

S2 Fig Oil Red O staining of diaphragm. Control = spontaneous ventilation without endotoxemia; MV = mechanical ventilation without sepsis; SV-LPS = endotoxemia with spontaneous ventilation; MV-LPS = endotoxemia with mechanical ventilation.
Bar scale = 50 μm.
(TIFF)

S3 Fig Confocal images of myosin heavy chain isoforms in diaphragm. Sections were stained with either mouse anti-slow myosin heavy chain (MyHC-1, in red) or anti-fast MyHC-2a (in green) and MyHC-2x (in blue). Cross-sectional areas of each fiber type were determined from immunofluorescence of MHC isoforms.
Control = spontaneous ventilation without endotoxemia; MV = mechanical ventilation without sepsis; SV-LPS = endotoxemia with spontaneous ventilation; MV-LPS = endotoxemia with mechanical ventilation.
Bar scale = 100 μm.
(TIFF)
