# Supplementary material for: Prolonged mechanical ventilation worsens sepsis-induced diaphragmatic dysfunction in the rat
Source: PLoS One. 2018 Aug 1;13(8):e0200429. doi: 10.1371/journal.pone.0200429 (PMC6070213; doi:10.1371/journal.pone.0200429)
Supplement: S1 Table — (PDF) [file pone.0200429.s001.pdf]

S1 Table. Arterial Blood Gases at 6 hours

	Control (n=8)	MV (n=8)	SV-LPS (n=8)	MV-LPS (n=8)
pH	ND	7.37 (7.32 – 7.41)	ND	7.32 (7.26 – 7.36)
$PaCO_2$, *mmHg*	ND	38 (28 – 47)	ND	40 (35 – 48)
PaO_2, *mmHg*	ND	83 (75 – 192)	ND	126 (104 – 148)
HCO_3^-, *mM*	ND	20.6 (16.5 – 24.6)	ND	20.2 (17.9 – 27.2)
Lactate, *mM*	ND	1.1 (0.8 – 1.6)	ND	2.1 (1.4 – 2.5)

Data are median (interquartile range). Control= spontaneous ventilation without endotoxemia; MV= mechanical ventilation without sepsis; SV-LPS= endotoxemia with spontaneous ventilation; MV-LPS= endotoxemia with mechanical ventilation; $PaCO_2$= arterial partial pressure of carbon dioxide; PaO_2= arterial partial pressure of oxygen; HCO_3^-= bicarbonate; ND=not determined.
