# Supplementary figures and images for: Prolonged mechanical ventilation worsens sepsis-induced diaphragmatic dysfunction in the rat
Source: PLoS One. 2018 Aug 1;13(8):e0200429. doi: 10.1371/journal.pone.0200429 (PMC6070213; doi:10.1371/journal.pone.0200429)

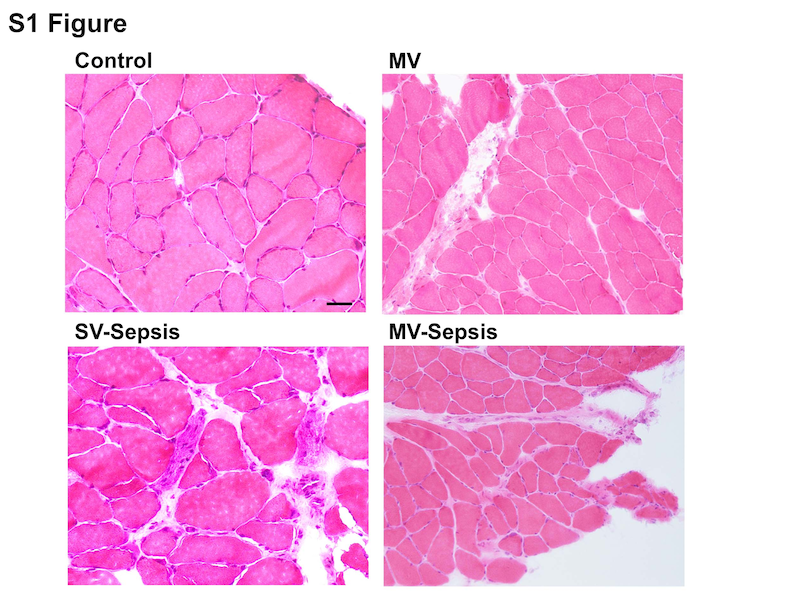

Supplement: S1 Fig — White arrows show inflammatory infiltrates. Control = spontaneous ventilation without endotoxemia; MV = mechanical ventilation without sepsis; SV-LPS = endotoxemia with spontaneous ventilation; MV-LPS = endotoxemia with mechanical ventilation. Bar scale = 50 μm. (TIFF) [file pone.0200429.s002.tiff]

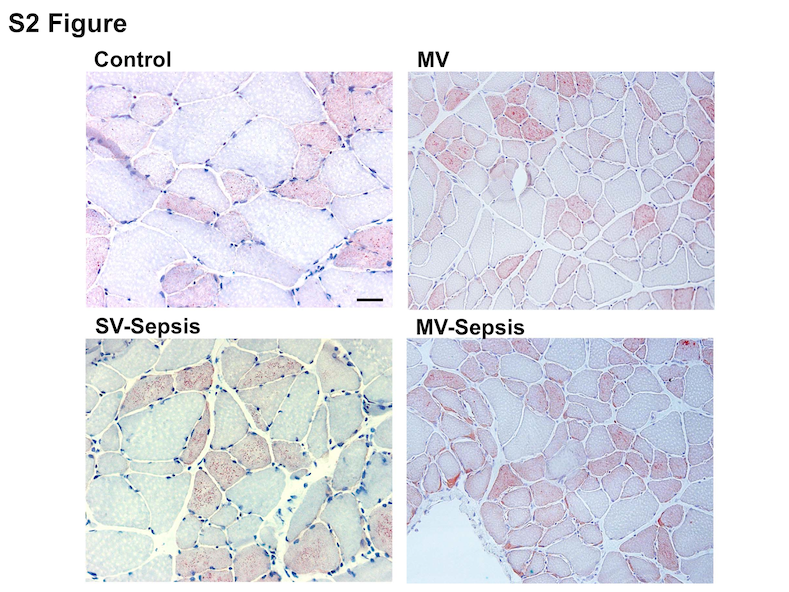

Supplement: S2 Fig — Control = spontaneous ventilation without endotoxemia; MV = mechanical ventilation without sepsis; SV-LPS = endotoxemia with spontaneous ventilation; MV-LPS = endotoxemia with mechanical ventilation. Bar scale = 50 μm. (TIFF) [file pone.0200429.s003.tiff]

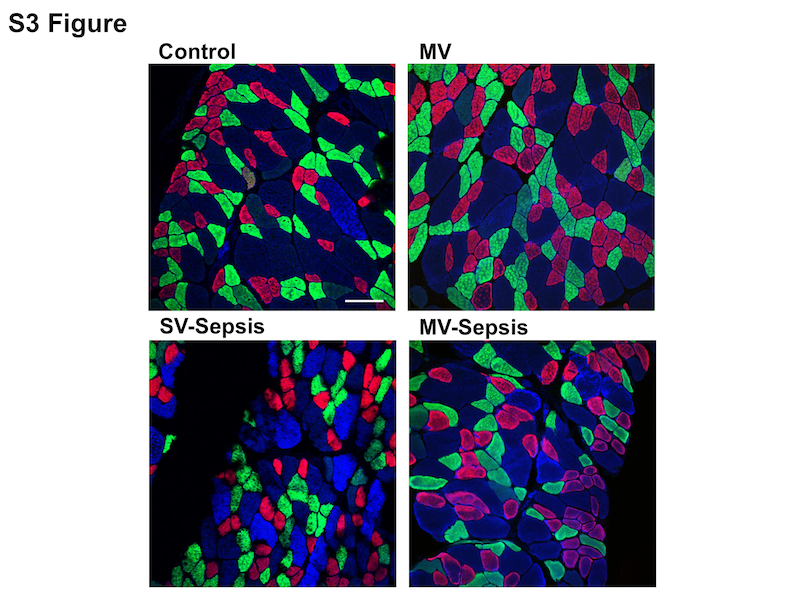

Supplement: S3 Fig — Sections were stained with either mouse anti-slow myosin heavy chain (MyHC-1, in red) or anti-fast MyHC-2a (in green) and MyHC-2x (in blue). Cross-sectional areas of each fiber type were determined from immunofluorescence of MHC isoforms. Control = spontaneous ventilation without endotoxemia; MV = mechanical ventilation without sepsis; SV-LPS = endotoxemia with spontaneous ventilation; MV-LPS = endotoxemia with mechanical ventilation. Bar scale = 100 μm. (TIFF) [file pone.0200429.s004.tiff]
